# Textbook outcomes in the laparoscopic common bile duct exploration of choledocholithiasis: a new comprehensive quality evaluation criterion

**DOI:** 10.3389/fsurg.2025.1623559

**Published:** 2025-08-14

**Authors:** Da Teng, Yue Xu

**Affiliations:** ^1^ Department of Hepatobiliary Pancreatic and Splenic Surgery Ward Ⅰ, The Affiliated ChuZhou Hospital of Anhui Medical University (The First People’s Hospital of ChuZhou), ChuZhou, China; ^2^ Department of Ultrasound Medicine, The Affiliated ChuZhou Hospital of Anhui Medical University (The First People’s Hospital of ChuZhou), ChuZhou, China

**Keywords:** choledocholithiasis, laparoscopic common bile duct exploration, textbook outcomes, surgery, prognosis

## Abstract

**Background:**

The textbook outcome (TO) is an innovative composite criterion that encompasses multiple perioperative events. It serves as a measure of perioperative quality and provides an objective reflection of the most desirable outcome. The concept of TO has been introduced to laparoscopic common bile duct exploration (LCBDE) to establish TO criteria and identify key risk factors associated with TO failure.

**Methods:**

Clinical data from 225 patients who underwent LCBDE for choledocholithiasis were retrospectively analyzed, categorizing them into “TO” and “TO-failure” groups based on whether TO was achieved. TO criteria were defined based on existing literature and the perioperative characteristics of LCBDE, including no residual stones, no bile leakage, no severe postoperative complications, no readmission or death within 30 days, and no extended hospitalization. The TO incidence rate was calculated, and univariate and multivariate logistic regression analyses were employed to identify perioperative characteristics and independent risk factors contributing to TO failure.

**Results:**

A total of 167 patients (74.2%) achieved TO. Independent risk factors for TO failure included ASA score ≥ 3 (OR: 9.260, 95% CI: 2.292–37.418, *P* = 0.002), T-tube drainage (TTD) (OR: 5.332, 95% CI: 1.625–17.497, *P* = 0.006), preoperative combined cholecystitis (OR: 3.448, 95% CI: 1.091–10.897, *P* = 0.035), preoperative combined cholangitis (OR: 11.468, 95% CI: 2.841–46.284, *P* = 0.001), and operative time ≥ 90 min (OR: 3.066, 95% CI: 1.253–7.503, *P* = 0.014).

**Conclusions:**

Applying the TO concept to LCBDE facilitates a more comprehensive and objective evaluation of perioperative characteristics in patients with choledocholithiasis. This approach contributes to the standardization of quality assessment in LCBDE, promoting the continuous improvement of surgical quality. Furthermore, achieving TO can enhance the overall quality of the healthcare system, potentially reducing healthcare costs. Additionally, TO aligns more closely with patient preferences, representing the optimal surgical outcome. As a holistic assessment tool, TO is poised to become a definitive quality standard for evaluating surgical procedures.

## Introduction

Choledocholithiasis is a prevalent and frequently encountered condition in clinical practice, with surgical intervention being a primary method for alleviating biliary obstruction and restoring normal biliary drainage. With the continuous advancements in laparoscopic techniques, laparoscopic common bile duct exploration (LCBDE) has gained widespread adoption globally. It offers benefits such as reduced trauma and quicker recovery and aligns with the principles of enhanced recovery after surgery (1). Traditional assessments of surgical quality, however, have often relied on single indicators such as mortality and complications. These singular outcome measures, particularly those based on low-probability events, fail to adequately capture the full perioperative experience, hinder improvements in medical care quality, and offer limited utility in predicting overall patient prognosis (2–4). Surgical quality significantly influences perioperative recovery and long-term outcomes. Consequently, objective evaluation of surgical quality is crucial for advancing clinical practices.

In this context, the textbook outcome (TO) concept has emerged as an innovative method for assessing perioperative prognostic criteria. Initially introduced by Kolfschoten et al. in 2013, this “all-or-none” patient-centered quality indicator consolidates multiple components to reflect the optimal outcome (5, 6). TO is valuable for evaluating patient-level outcomes, designating medical centers, assessing hospital performance, and monitoring surgical quality. It has been linked to enhanced long-term survival rates and reduced healthcare costs and is currently applied to various benign and malignant conditions (7–9). To date, no studies have described the TO for LCBDE, and specific evaluation criteria remain absent. Incorporating the TO concept into the quality assessment of LCBDE is a valuable strategy that addresses the limitations of single-criteria evaluations, providing a comprehensive perioperative prognostic benchmark. Additionally, it offers the advantages of simplicity, ease of implementation, and broad applicability. Therefore, this cohort study aims to establish TO criteria for LCBDE in the treatment of choledocholithiasis and identify the risk factors contributing to TO failure, to enhance the quality of perioperative care in LCBDE.

## Materials and methods

### Study design and patient population

This cohort study included clinical data from 225 patients diagnosed with choledocholithiasis who underwent LCBDE at The Affiliated ChuZhou Hospital of Anhui Medical University between January 2019 and June 2024. The study was conducted in accordance with the Declaration of Helsinki and was approved by the Institutional Ethics Committee of The Affiliated ChuZhou Hospital of Anhui Medical University [approval code, 2024 Ethical Review (Bio) No. 35; approval date, 23 October 2024]. Given the retrospective nature of this study, and since no additional examinations beyond those necessary for patient care were required during the perioperative period, patients were informed upon admission that their clinical data might be used for research purposes. Data were anonymized, and patient confidentiality was maintained throughout the study. The Institutional Ethics Committee approved the use of patient data in this research.

### Inclusion and exclusion criteria

Inclusion criteria: (1) confirmed diagnosis of choledocholithiasis through imaging examinations, such as abdominal ultrasound, computed tomography scans, and magnetic resonance cholangiopancreatography, (2) provided signed informed consent for the surgery. (3) were aged 18 years or older at the time of surgery, and (4) any pre-existing biliary inflammation controlled with anti-inflammatory and symptomatic treatments prior to the surgery.

Exclusion criteria: (1) incomplete clinical data, (2) emergency surgery for acute obstructive suppurative cholangitis (AOSC), (3) presence of hepatolithiasis, (4) preoperative biliary inflammation that had been treated with invasive procedures such as percutaneous transhepatic cholangiography (PTC) or endoscopic retrograde cholangiopancreatography (ERCP), and (5) inability to tolerate surgery due to comorbid conditions.

### Data collection and definition

Perioperative data were collected, including age, gender, body mass index (BMI), hypertension, diabetes, history of biliary disease (intrahepatic choledocholithiasis, biliary stenosis/dilatation, etc.), history of malignant tumor treatment, American Society of Anesthesiologists (ASA) score, Barthel index, common bile duct (CBD) treatment method [primary duct closure (PDC) or T-tube drainage (TTD)], preoperative combined cholecystitis, preoperative combined cholangitis, preoperative biochemical findings [white blood cell (WBC) count, C-reactive protein (C-RP), albumin (ALB), total bilirubin (TBIL), alanine aminotransferase (ALT), and aspartate transaminase (AST)], number of stones, maximum stone diameter, CBD diameter, operation time, estimated blood loss, postoperative complications (hemorrhage, bile leakage, pancreatitis, cholangitis, electrolyte imbalance), hospital stay, and medical costs. The ASA classification and grading system was used as defined by the American Society of Anesthesiologists (10). The Barthel index was assessed based on patient mobility and self-care, with scores ranging from 0 to 100 (11). Postoperative complications were graded using the Clavien–Dindo classification to determine severity (12). Residual stones were defined as stones that were incompletely removed during the procedure. Bile leakage was characterized by an elevated bilirubin concentration in the abdominal drainage fluid or intra-abdominal fluid of at least three times the serum bilirubin level, measured on or after the third postoperative day, or the need for re-intervention (e.g., interventional drainage or laparotomy) due to bile collection caused by choleperitonitis. The severity of the leakage was graded according to the International Study Group of Liver Surgery (13).

### Operative techniques

Routine tracheal intubation was performed for general anesthesia. Once anesthesia was established, the patient was positioned supine, and carbon dioxide pneumoperitoneum was created. The four-trocar technique was used, with 10 mm ports for the camera and epigastric port, and 5 mm ports for the remaining access sites. Cholecystectomy was performed first, followed by exposure of the hepatoduodenal ligament. Electrocoagulation was used to longitudinally open the membrane structure, exposing the anterior wall of the CBD. The posterior longitudinal incision of the CBD was confirmed. A flexible choledochoscope (Olympus, Tokyo, Japan) was then inserted to explore the biliary system, from the intrahepatic secondary bile ducts to the medial papillary portion of the duodenal wall. A mesh basket was employed to remove the stones. In cases with PDC, a 4-0 absorbable suture (Vicryl, Ethicon Inc., Somerville, NJ, USA) was used to close the anterior wall of the CBD. For TTD, a T-tube (size 18−22^#^) was placed through the anterior wall incision of the CBD, secured with interrupted sutures, and the tube was exited from the body. A Winslow's foramen drainage tube was routinely placed. All surgeries were performed by the same surgical team.

### Definition of TO and follow-up

TO is a composite measure that incorporates several criteria to reflect the optimal perioperative outcome. Based on prior studies of other surgical procedures and the perioperative characteristics following LCBDE, the TO criteria were defined as follows: (1) no residual stones, (2) no bile leakage, (3) no severe postoperative complications, (4) no readmission or death within 30 days, and (5) no extended hospitalization. Severe postoperative complications were defined as Clavien–Dindo classification ≥ III, and prolonged hospitalization was defined as exceeding the 75th percentile of the total hospital stay duration within the cohort (2, 14). TO was considered achieved if all these criteria were met. The median follow-up period for patients was 31 months (range, 8–66 months).

### Statistical analysis

Statistical analysis was performed using IBM SPSS 22.0 (Chicago, IL, USA). Categorical data were presented as counts and percentages [*N* (%)], with comparisons made using the chi-square test (*χ*^2^ test). The Kolmogorov–Smirnov *Z*-test was used to assess the normality of distribution for each group. Data not following a normal distribution were expressed as *M* (*P*_25_, *P*_75_), and comparisons between two groups were performed using the Mann–Whitney *U*-test. Univariate and multivariate logistic regression analyses were conducted to identify independent risk factors for TO failure. The results were presented as odds ratios (OR) with 95% confidence intervals (*CI*). A *P*-value of <0.05 was considered statistically significant.

## Results

### Comparison of baseline information and clinical characteristics

**Figure 1:**
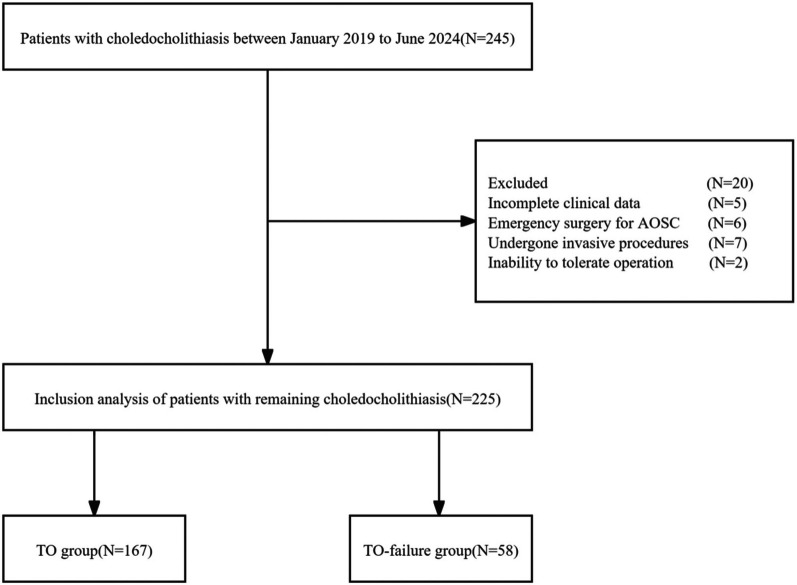
Flowchart of the participant population.

**Figure 2:**
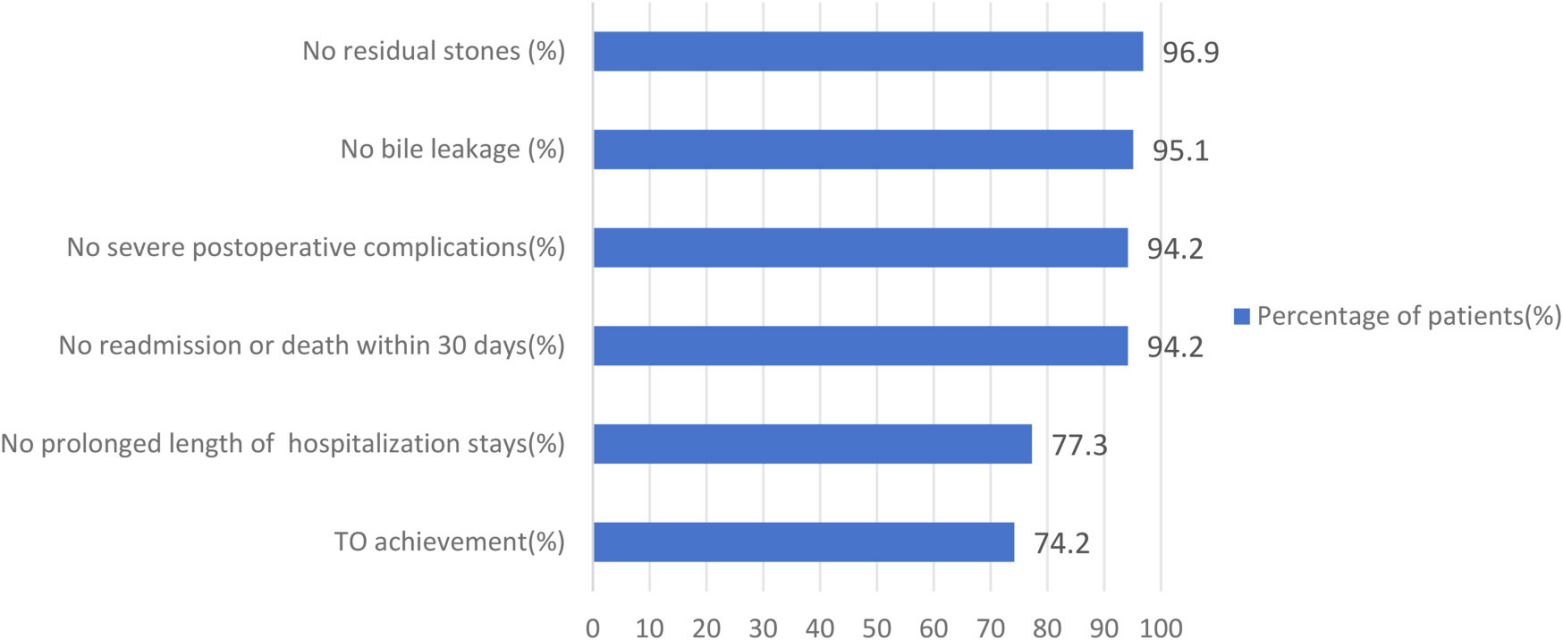
Distribution of TO criteria in the study cohort.

A total of 225 patients were included in this study (Figure 1). The cohort consisted of 106 males (47.1%) and 119 females (52.9%), with ages ranging from 18–91 years and a median age of 58 years. The study results revealed that 218 patients (96.9%) had no residual stones, 214 patients (95.1%) had no bile leakage, 212 patients (94.2%) experienced no severe postoperative complications, 212 patients (94.2%) had no readmissions or deaths, and 174 patients (77.3%) had no prolonged hospital stays. Patients were divided into two groups based on whether they achieved TO: the TO group, with 167 patients (74.2%), and the TO-failure group, with 58 patients (25.8%). No significant differences were found between the two groups with respect to baseline characteristics, such as age, gender, BMI, hypertension, diabetes, and history of malignant tumor treatment (*P* > 0.05). However, significant differences were observed in perioperative clinical characteristics. The TO-failure group had a higher percentage of patients with a history of biliary disease (39.7% vs. 20.4%, *P* = 0.004); an ASA score ≥ 3 (44.8% vs. 4.8%, *P* < 0.001); a lower Barthel index [90 (80–96.25) vs. 100 (95–100), *P* < 0.001]; a higher proportion of cholecystitis (65.5% vs. 34.1%, *P* < 0.001) and cholangitis (39.7% vs. 3.6%, *P* < 0.001); higher levels of WBC [8.84 (6.31–14.73) vs. 6.67 (5.25–9.51) × 10^9^/L, *P* = 0.001], C-RP [31.87 (2.96–122.55) vs. 3.58 (1.54–22.64) mg/L, *P* < 0.001], TBIL [59.05 (40.98–95.40) vs. 45.20 (22.40–70.50) μmol/L, *P* = 0.005], and AST [170.65 (72.01–336.39) vs. 100.54 (33.55–244.20) U/L, *P* = 0.012]; and lower ALB [39.50 (35.10–42.53) vs. 42.50 (39.50–45.10) g/L, *P* < 0.001]. The treatment method for the CBD also differed significantly between the groups, with a higher proportion of TTD compared with PDC in the TO-failure group (*P* < 0.001). Additionally, the TO-failure group had a larger maximum stone diameter [8 (5–10) vs. 6 (5–8) mm, *P* = 0.019], longer operation time [110 (77.75–160) vs. 85 (69–111) min, *P* < 0.001], and higher estimated blood loss [35 (20–50) vs. 20 (10–30) ml, *P* < 0.001]. A total of 31 patients (13.8%) experienced postoperative complications, graded according to the Clavien–Dindo classification: 9 (4%) patients were grade I, 9 (4%) patients were grade II, and 13 (5.8%) patients were grade IIIa. The TO-failure group also had longer hospital stays [14 (12.75–15) vs. 7 (6–9) days, *P* < 0.001] and higher medical costs [26,327.46 (19,704.05–35,740.96) vs. 16,494.58 (14,877.94–18,649.46) CNY, *P* < 0.001], as shown in Figure 2 and Table 1.

**Table 1:** Comparison of perioperative variables between TO and TO failure groups.

Variables	Total (*n* = 225)	TO group (*n* = 167)	TO-failure group (*n* = 58)	P-value
Age (years)	58 (49–70)	58 (48–70)	59 (49–70.50)	0.442
Sex (male/female, *n*%)	106 (47.1)/119 (52.9)	76 (45.5)/91 (54.5)	30 (51.7)/28 (48.3)	0.414
BMI (kg/m^2^)	23.54 (22.63–24.47)	23.45 (22.58–24.20)	23.64 (22.84–24.60)	0.052
Hypertension (yes/no, *n*%)	53 (23.6)/172 (76.4)	39 (23.4)/128 (76.6)	14 (24.1)/44 (75.9)	0.903
Diabetes (yes/no, *n*%)	24 (10.7)/201 (89.3)	15 (9.0)/152 (91.0)	9 (15.5)/49 (84.5)	0.165
History of biliary disease (yes/no, *n*%)	57 (25.3)/168 (74.7)	34 (20.4)/133 (79.6)	23 (39.7)/35 (60.3)	0.004
History of malignant tumor treatment (yes/no, *n*%)	17 (7.6)/208 (92.4)	11 (6.6)/156 (93.4)	6 (10.3)/52 (89.7)	0.351
ASA score (1–2/≥3, *n*%)	191 (84.9)/34 (15.1)	159 (95.2)/8 (4.8)	32 (55.2)/26 (44.8)	<0.001
The Barthel index score	95 (90–100)	100 (95–100)	90 (80–96.25)	<0.001
The treatment method of CBD (PDC/TTD, *n*%)	109 (48.4)/116 (51.6)	96 (57.5)/71 (42.5)	13 (22.4)/45 (77.6)	<0.001
Preoperative combined cholecystitis (yes/no, *n*%)	95 (42.2)/130 (57.8)	57 (34.1)/110 (65.9)	38 (65.5)/20 (34.5)	<0.001
Preoperative combined cholangitis (yes/no, *n*%)	29 (12.9)/196 (87.1)	6 (3.6)/161 (96.4)	23 (39.7)/35 (60.3)	<0.001
Preoperative biochemistry findings				
WBC (×109/L)	7.21 (5.41–10.97)	6.67 (5.25–9.51)	8.84 (6.31–14.73)	0.001
C-RP (mg/L)	4.37 (1.61–33.53)	3.58 (1.54–22.64)	31.87 (2.96–122.55)	<0.001
ALB (g/L)	41.60 (38.60–44.60)	42.50 (39.50–45.10)	39.50 (35.10–42.53)	<0.001
TBIL (μmol/L)	49.20 (25.65–75.50)	45.20 (22.40–70.50)	59.05 (40.98–95.40)	0.005
ALT (U/L)	152.20 (59.60–335.85)	140 (50.70–314.90)	199.85 (83.65–361.18)	0.090
AST (U/L)	120.20 (43.15–300.38)	100.54 (33.55–244.20)	170.65 (72.01–336.39)	0.012
Number of stones (single/multiple, *n*%)	120 (53.3)/105 (46.7)	95 (56.9)/72 (43.1)	25 (43.1)/33 (56.9)	0.070
The maximum diameter of stone (mm)	6 (5–8)	6 (5–8)	8 (5–10)	0.019
Diameter of CBD (mm)	12 (10–15)	12 (10–15)	14.5 (10–20)	0.054
Operation time (min)	88 (70–121)	85 (69–111)	110 (77.75–160)	<0.001
Estimated blood loss(ml)	20 (10–40)	20 (10–30)	35 (20–50)	<0.001
Total postoperative complications (yes/no, *n*%)	31 (13.8)/194 (86.2)	9 (5.4)/158 (94.6)	22 (37.9)/36 (62.1)	<0.001
Postoperative complications Clavien–Dindo ≤ II (yes/no, *n*%)	18 (8.0)/207 (92)	9 (5.4)/158 (94.6)	9 (15.5)/49 (84.5)	0.014
Hemorrhage (yes/no, *n*%)	1 (0.4)/224 (99.6)	0 (0)/167 (100)	1 (1.7)/57 (98.3)	0.258
Pancreatitis (yes/no, *n*%)	2 (0.9)/223 (99.1)	2 (1.2)/165 (98.8)	0 (0)/58 (100)	1.000
Cholangitis (yes/no, *n*%)	13 (5.8)/212 (94.2)	3 (1.8)/164 (98.2)	10 (17.2)/48 (82.8)	<0.001
Electrolyte disorder (yes/no, *n*%)	7 (3.1)/218 (96.9)	5 (3.0)/162 (97.0)	2 (3.4)/56 (96.6)	1.000
Hospital stays (days)	8 (6.5–11)	7 (6–9)	14 (12.75–15)	<0.001
Medical costs (CNY)	17,398.09(15,488.88–20,728.66)	16,494.58(14,877.94–18,649.46)	26,327.46(19,704.05–35,740.96)	<0.001

### TO and independent risk factors associated with TO failure

Table 2 summarizes the univariate and multivariate logistic regression analysis results for factors contributing to TO failure in LCBDE. The univariate analysis identified several factors associated with TO failure, including history of biliary disease, ASA score, Barthel index, CBD treatment method, preoperative cholecystitis and cholangitis, WBC, C-RP, ALB, TBIL, stone diameter, CBD diameter, operation time, and estimated blood loss. Multivariate analysis revealed the following independent risk factors for TO failure: ASA score ≥ 3 (OR: 9.260, 95% CI: 2.292–37.418, *P* = 0.002), TTD (OR: 5.332, 95% CI: 1.625–17.497, *P* = 0.006), preoperative combined cholecystitis (OR: 3.448, 95% CI: 1.091–10.897, *P* = 0.035), preoperative combined cholangitis (OR: 11.468, 95% CI: 2.841–46.284, *P* = 0.001), and operative time ≥ 90 min (OR: 3.066, 95% CI: 1.253–7.503, *P* = 0.014).

**Table 2:** Univariate and multivariate logistic regression analyses of risk factors associated with achieving textbook outcome undergoing LCBDE for patients with choledocholithiasis.

Variables	OR comparison	UV OR (95% CI)	UV *P-*value	MV OR (95% CI)	MV *P-*value
Age (years)	<65/≥65	1.085 (0.588–2.001)	0.794		
Sex	Male/female	0.779 (0.429–1.418)	0.414		
BMI (kg/m^2^)	<24/≥24	1.782 (0.951–3.336)	0.071		
Hypertension	No/yes	1.044 (0.519–2.103)	0.903		
Diabetes	No/yes	1.861 (0.767–4.518)	0.170		
History of biliary disease	No/yes	2.571 (1.346–4.909)	0.004	NA	0.347
History of malignant tumor treatment	No/yes	1.636 (0.577–4.644)	0.355		
ASA score	1–2/≥3	16.148 (6.707–38.882)	<0.001	9.260 (2.292–37.418)	0.002
The Barthel index	<85/≥85	0.063 (0.022–0.180)	<0.001	NA	0.136
The treatment method of CBD	PDC/TTD	4.680 (2.349–9.324)	<0.001	5.332 (1.625–17.497)	0.006
Preoperative combined cholecystitis	No/yes	3.667 (1.955–6.877)	<0.001	3.448 (1.091–10.897)	0.035
Preoperative combined cholangitis	No/yes	17.633 (6.685–46.515)	<0.001	11.468 (2.841–46.284)	0.001
Preoperative biochemistry findings					
WBC (×109/L)	<10/≥10	3.060 (1.626–5.758)	0.001	NA	0.453
C-RP (mg/L)	<10/≥10	3.388 (1.810–6.343)	<0.001	NA	0.593
ALB (g/L)	<35/≥35	0.097 (0.033–0.284)	<0.001	NA	0.113
TBIL (μmol/L)	<34.2/≥34.2	2.437 (1.223–4.858)	0.011	NA	0.278
ALT (U/L)	<100/≥100	1.873 (0.964–3.641)	0.064		
AST (U/L)	<100/≥100	1.659 (0.897–3.071)	0.107		
Number of stones	Single/multiple	1.742 (0.953–3.184)	0.071		
The maximum diameter of stone (mm)	<10/≥10	3.048 (1.464–6.345)	0.003	NA	0.431
Diameter of CBD (mm)	<15/≥15	2.036 (1.109–3.738)	0.022	NA	0.565
Operation time (min)	<90/≥90	3.005 (1.592–5.671)	0.001	3.066 (1.253–7.503)	0.014
Estimated blood loss (ml)	<30/≥30	3.869 (2.060–7.267)	<0.001	NA	0.659

## Discussion

Traditionally, perioperative prognostic assessments have typically relied on single outcome analyses for evaluating patient recovery (15). However, a singular outcome is insufficient to fully assess recovery or to capture the multidimensional and individualized aspects of perioperative care (16). TO integrates various perioperative outcomes, deeming the prognosis favorable only when all criteria are met. As a more comprehensive, objective, and patient-centered evaluation tool, TO enhances the likelihood of predicting favorable patient outcomes. Since its introduction, TO has been applied across various surgical contexts, continuously refined for definition and validation (17, 18). As a composite measure, TO also serves as a valuable tool for assessing overall hospital performance, providing specific benchmarks for quality improvement based on individual outcomes (19). Despite its widespread use, however, specific TO evaluation criteria for choledocholithiasis treated with LCBDE remain lacking, and further analysis and validation of influencing factors are still necessary.

In this study, criteria for TO of LCBDE were proposed based on both previous retrospective studies and our clinical experience. Unlike prior studies, this study included “no residual stones” and “no bile leakage” as part of the evaluation, considering the disease-specific perspective. For choledocholithiasis treatment, the primary objective is the complete removal of stones and the restoration of smooth biliary drainage. Retained stones signify a failure of surgical treatment, while bile leakage is a significant complication. It is one of the most critical safety indicators post-LCBDE, as the procedure often necessitates an incision in the anterior wall of the CBD, compromising its structural and functional integrity. Studies have shown bile leakage incidence following LCBDE to range from 1.6%–11.3%, with the potential to directly lead to further postoperative complications (20–22). Consequently, these factors were deemed essential components of the postoperative TO criteria for LCBDE.

TO failure indicates a poor postoperative prognosis, often resulting from perioperative complications, prolonged hospitalization, or readmissions. The causes of TO failure are multifactorial, involving not only surgical and patient-specific factors but also external elements such as cultural norms, healthcare payment schemes, and access to long-term rehabilitation facilities (23). A retrospective study on health economics found that patients who failed to achieve TO incurred a relative increase in variable costs exceeding 95% compared with those who met the TO criteria (24). Identifying risk factors for TO failure after LCBDE is therefore crucial, offering benefits to healthcare providers, patients, and hospital management alike. Our findings highlight the importance of the ASA score, a widely used system for preoperative status assessment that helps healthcare teams quickly evaluate a patient's physical condition. This enables rational decision-making regarding surgical, anesthetic, and perioperative management. Elevated ASA scores increase the likelihood of postoperative complications and are strongly associated with higher mortality (25, 26). Moreover, higher ASA scores are significant predictors of prolonged hospitalization and readmissions (25, 27). A systematic review indicated that higher ASA scores negatively impact the likelihood of achieving TO in surgical procedures (28). Specifically, in hepatobiliary surgery, Clocchiatti et al. (29) found that an ASA score ≥ 3 reduced the chances of achieving TO following surgery for perihilar cholangiocarcinoma. Similarly, Lucocq et al. (30) concluded that an ASA score ≥ 3 increased the likelihood of TO failure when evaluating the overall quality of elective laparoscopic cholecystectomy. Therefore, a high preoperative ASA score signals greater risk, a higher incidence of perioperative adverse events, and a negative impact on recovery, ultimately influencing the achievement of TO.

Most patients with choledocholithiasis undergo surgery only after clinical symptoms manifest, with some delaying treatment to the point of requiring emergency surgery due to AOSC or hepatic dysfunction (31). Additionally, obstructive jaundice leads to cholestasis, coagulation disorders, and increased risks of perioperative biliary infections, which further impair hepatic regenerative function and are strongly linked to pro-inflammatory responses (32). From a surgical perspective, preoperative combined cholecystitis and cholangitis, particularly in areas such as Calot's triangle and the hepatoduodenal ligament, often lead to adhesion or fibrosis. These acute or chronic inflammatory conditions increase surgical difficulty, elevate the risk of intraoperative hemorrhage and bile duct injury, prolong operative time, and negatively affect patient prognosis. Perioperative recovery is significantly influenced by the presence of obstructive jaundice and hepatic insufficiency, which are characterized by hepatic anabolic and metabolic dysfunction, as well as activation of the inflammatory response. These factors increase the likelihood of postoperative complications, including hemorrhage, infections, and bile leakage. Our study found that preoperative combined cholecystitis and cholangitis contributed to an elevated risk of TO failure. In patients with acute cholecystitis lasting more than 3 days (33), acute cholangitis, AOSC, and hepatic dysfunction, alternative treatment options such as PTC or ERCP may be considered to control biliary inflammation, alleviate biliary obstruction, and facilitate stone removal to mitigate the risk of TO failure.

The T-tube plays a critical role in providing biliary support and facilitating bile drainage, which helps maintain the internal diameter of the CBD, reduces postoperative biliary pressure, and aids in the retrieval of stones during the postoperative period (34, 35). However, the loss of bile, a key digestive fluid, can disrupt perioperative digestive function and compromise the stability of the internal environment. Additionally, the external catheter is susceptible to biliary infections, bile leakage, and other complications. Furthermore, the formation of a sinusoidal tract is closely linked to the patient's physical condition and nutritional status, and in some cases, the T-tube may become detached (36–38). From the perspective of CBD closure, several studies have highlighted the advantages of PDC over TTD in optimizing perioperative outcomes and enhancing patient recovery. Jiang et al. (39) concluded that PDC resulted in shorter operative times, reduced hospital stays, and fewer instances of residual stones. Zhuang et al. (40) found that patients undergoing PDC experienced faster gastrointestinal recovery and shorter hospitalizations compared with those with TTD. Consistent with these findings, the present study demonstrates that PDC is more likely to lead to the achievement of TO compared with TTD. During perioperative recovery, bile can be fully discharged into the intestine, aiding in digestion, maintaining the stability of the bile-acid intestinal-liver circulation, and preventing fluid loss. Moreover, as the biliary system is sealed from external contact, the risk of postoperative biliary infections is minimized. The patient also experiences greater comfort and convenience, with improved compliance and fewer adverse effects such as postoperative pain. Therefore, PDC is more advantageous than TTD in optimizing the surgical process, reducing operative trauma, and promoting rapid recovery. These benefits also significantly enhance the likelihood of achieving TO following LCBDE.

This study has several limitations. Firstly, as a single-center cohort study, its findings may not be generalizable to other diseases or healthcare institutions. Secondly, the relatively small sample size introduces potential selection bias, which needs to be further examined through larger multicenter studies. Finally, the current TO criteria lack robust evidence-based validation and may evolve as medical practices and knowledge advance.

## Conclusions

In conclusion, the proposed TO criteria for LCBDE encompass the absence of residual stones, no bile leakage, no severe postoperative complications, no readmission or mortality within 30 days, and no prolonged hospitalization. The TO achievement rate was 74.2%, and attaining TO has been shown to correlate with a more favorable prognosis. Independent risk factors for TO failure include ASA score ≥3, TTD, preoperative combined cholecystitis, preoperative combined cholangitis, and operative time ≥ 90 min. The adoption of the TO concept in LCBDE offers a more comprehensive and objective evaluation of perioperative outcomes in patients with choledocholithiasis. It supports the standardization of LCBDE quality assessments, thereby fostering continuous improvements in surgical practices. Additionally, the attainment of TO contributes to the overall enhancement of healthcare quality and has the potential to reduce healthcare costs. Furthermore, the TO concept aligns closely with patient preferences, representing the optimal surgical outcome. As a holistic metric, TO is poised to become a definitive quality standard for evaluating surgical procedures.

## Data Availability

The original contributions presented in the study are included in the article/Supplementary Material; further inquiries can be directed to the corresponding author.

## References

[1] Zhang Z Shao G Li Y Li K Zhai G Dang X Efficacy and safety of laparoscopic common bile duct exploration with primary closure and intraoperative endoscopic nasobiliary drainage for choledocholithiasis combined with cholecystolithiasis. Surg Endosc. (2023) 37(3):1700–9. 10.1007/s00464-022-09601-3 36207648 PMC10017613

[2] Zhang XJ Fei H Guo CG Sun CY Li ZF Li Z Analysis of textbook outcomes for ampullary carcinoma patients following pancreaticoduodenectomy. World J Gastrointest Surg. (2023) 15(10):2259–71. 10.4240/wjgs.v15.i10.2259 37969713 PMC10642474

[3] Parina RP Chang DC Rose JA Talamini MA. Is a low readmission rate indicative of a good hospital? J Am Coll Surg. (2015) 220(2):169–76. 10.1016/j.jamcollsurg.2014.10.020 25529903

[4] Abbott TEF Fowler AJ Dobbs TD Harrison EM Gillies MA Pearse RM. Frequency of surgical treatment and related hospital procedures in the UK: a national ecological study using hospital episode statistics. Br J Anaesth. (2017) 119(2):249–57. 10.1093/bja/aex137 28854546

[5] Kolfschoten NE Kievit J Gooiker GA van Leersum NJ Snijders HS Eddes EH Focusing on desired outcomes of care after colon cancer resections; hospital variations in ‘textbook outcome’. Eur J Surg Oncol. (2013) 39(2):156–63. 10.1016/j.ejso.2012.10.007 23102705

[6] Merath K Chen Q Bagante F Beal E Akgul O Dillhoff M Textbook outcomes among medicare patients undergoing hepatopancreatic surgery. Ann Surg. (2020) 271(6):1116–23. 10.1097/SLA.0000000000003105 30499800

[7] Yang T Liu DQ Qiu W Fan ZQ Sun LY Wang NY The Barthel index predicts surgical textbook outcomes following hepatectomy for elderly patients with hepatocellular carcinoma: a multicenter cohort study. Am J Surg. (2024) 237:115761. 10.1016/j.amjsurg.2024.05.002 38777717

[8] de Graaff MR Hendriks TE Wouters M Nielen M de Hingh I Koerkamp BG Assessing quality of hepato-pancreato-biliary surgery: nationwide benchmarking. Br J Surg. (2024) 111(5):znae119. 10.1093/bjs/znae119 38747683 PMC11095128

[9] Iseda N Iguchi T Itoh S Sasaki S Honboh T Yoshizumi T Textbook outcome in the laparoscopic cholecystectomy of acute cholecystitis. Asian J Endosc Surg. (2023) 16(4):741–6. 10.1111/ases.13238 37525942

[10] Irlbeck T Zwißler B Bauer A. ASA klassifikation: wandel im laufe der zeit und darstellung in der literatur [ASA classification: transition in the course of time and depiction in the literature]. Anaesthesist. (2017) 66(1):5–10. 10.1007/s00101-016-0246-4 27995282

[11] Sainsbury A Seebass G Bansal A Young JB. Reliability of the Barthel index when used with older people. Age Ageing. (2005) 34(3):228–32. 10.1093/ageing/afi063 15863408

[12] Clavien PA Barkun J de Oliveira ML Vauthey JN Dindo D Schulick RD The Clavien-Dindo classification of surgical complications: five-year experience. Ann Surg. (2009) 250(2):187–96. 10.1097/SLA.0b013e3181b13ca2 19638912

[13] Koch M Garden OJ Padbury R Rahbari NN Adam R Capussotti L Bile leakage after hepatobiliary and pancreatic surgery: a definition and grading of severity by the International Study Group of Liver Surgery. Surgery. (2011) 149(5):680–8. 10.1016/j.surg.2010.12.002 21316725

[14] Xu FQ Ye TW Wang DD Xie YM Zhang KJ Cheng J Association of preoperative albumin-bilirubin with surgical textbook outcomes following laparoscopic hepatectomy for hepatocellular carcinoma. Front Oncol. (2022) 12:964614. 10.3389/fonc.2022.964614 35965571 PMC9373871

[15] Ruzzenente A Poletto E Conci S Campagnaro T Valle BD De Bellis M Factors related to textbook outcome in laparoscopic liver resections: a single western centre analysis. J Gastrointest Surg. (2022) 26(11):2301–10. 10.1007/s11605-022-05413-x 35962214 PMC9643260

[16] Denbo J Anaya DA. Textbook outcomes following liver resection for cancer: a new standard for quality benchmarking and patient decision making. Ann Surg Oncol. (2020) 27(9):3118–20. 10.1245/s10434-020-08550-2 32385768

[17] D’Silva M Cho JY Han HS Yoon YS Lee HW Lee JS The value of analyzing textbook outcomes after laparoscopic hepatectomy-a narrative review. Transl Cancer Res. (2023) 12(3):631–7. 10.21037/tcr-22-2122 37033349 PMC10080324

[18] Naumann DN Bhangu A Brooks A Martin M Cotton BA Khan M A call for patient-centred textbook outcomes for emergency surgery and trauma. Br J Surg. (2022) 109(12):1191–3. 10.1093/bjs/znac271 35979582 PMC10364760

[19] Munir MM Alaimo L Moazzam Z Endo Y Lima HA Shaikh C Textbook oncologic outcomes and regionalization among patients undergoing hepatic resection for intrahepatic cholangiocarcinoma. J Surg Oncol. (2023) 127(1):81–9. 10.1002/jso.27102 36136327 PMC10087698

[20] El-Geidie AA. Is the use of T-tube necessary after laparoscopic choledochotomy? J Gastrointest Surg. (2010) 14(5):844–8. 10.1007/s11605-009-1133-y 20232173

[21] Koc B Karahan S Adas G Tutal F Guven H Ozsoy A. Comparison of laparoscopic common bile duct exploration and endoscopic retrograde cholangiopancreatography plus laparoscopic cholecystectomy for choledocholithiasis: a prospective randomized study. Am J Surg. (2013) 206(4):457–63. 10.1016/j.amjsurg.2013.02.004 23871320

[22] Liu D Cao F Liu J Xu D Wang Y Li F. Risk factors for bile leakage after primary closure following laparoscopic common bile duct exploration: a retrospective cohort study. BMC Surg. (2017) 17(1):1. 10.1186/s12893-016-0201-y 28056934 PMC5217550

[23] Pretzsch E Koliogiannis D D'Haese JG Ilmer M Guba MO Angele MK Textbook outcome in hepato-pancreato-biliary surgery: systematic review. BJS Open. (2022) 6(6):zrac149. 10.1093/bjsopen/zrac149 36449597 PMC9710735

[24] Jacobs MA Kim J Tetley JC Schmidt S Brimhall BB Mika V Cost of failure to achieve textbook outcomes: association of insurance type with outcomes and cumulative cost for inpatient surgery. J Am Coll Surg. (2023) 236(2):352–64. 10.1097/XCS.0000000000000468 36648264 PMC11549895

[25] Meyer AC Eklund H Hedström M Modig K. The ASA score predicts infections, cardiovascular complications, and hospital readmissions after hip fracture-A nationwide cohort study. Osteoporos Int. (2021) 32(11):2185–92. 10.1007/s00198-021-05956-w 34013459 PMC8563539

[26] Xu SJ Lin LQ Chen C Chen TY You CX Chen RQ Textbook outcome after minimally invasive esophagectomy is an important prognostic indicator for predicting long-term oncological outcomes with locally advanced esophageal squamous cell carcinoma. Ann Transl Med. (2022) 10(4):161. 10.21037/atm-22-506 35280418 PMC8908120

[27] Tran A Mai T El-Haddad J Lampron J Yelle JD Pagliarello G Preinjury ASA score as an independent predictor of readmission after major traumatic injury. Trauma Surg Acute Care Open. (2017) 2(1):e000128. 10.1136/tsaco-2017-000128 29766118 PMC5887763

[28] Carbonell-Morote S Yang HK Lacueva J Rubio-García JJ Alacan-Friedrich L Fierley L Textbook outcome in oncological gastric surgery: a systematic review and call for an international consensus. World J Surg Oncol. (2023) 21(1):288. 10.1186/s12957-023-03166-8 37697286 PMC10496160

[29] Clocchiatti L Marino R Ratti F Pedica F Casadei Gardini A Lorenzin D Defining and predicting textbook outcomes for perihilar cholangiocarcinoma: analysis of factors improving achievement of desired postoperative outcomes. Int J Surg. (2024) 110(1):209–18. 10.1097/JS9.0000000000000793 37800550 PMC10793762

[30] Lucocq J Scollay J Patil P. Evaluation of textbook outcome as a composite quality measure of elective laparoscopic cholecystectomy. JAMA Netw Open. (2022) 5(9):e2232171. 10.1001/jamanetworkopen.2022.32171 36125810 PMC9490496

[31] Xiao LK Xiang JF Wu K Fu X Zheng MY Song XX The reasonable drainage option after laparoscopic common bile duct exploration for the treatment of choledocholithiasis. Clin Res Hepatol Gastroenterol. (2018) 42(6):564–9. 10.1016/j.clinre.2018.07.005 30145281

[32] Zhang XF Beal EW Merath K Ethun CG Salem A Weber SM Oncologic effects of preoperative biliary drainage in resectable hilar cholangiocarcinoma: percutaneous biliary drainage has no adverse effects on survival. J Surg Oncol. (2018) 117(6):1267–77. 10.1002/jso.24945 29205351

[33] Yokoe M Hata J Takada T Strasberg SM Asbun HJ Wakabayashi G Tokyo guidelines 2018: diagnostic criteria and severity grading of acute cholecystitis (with videos). J Hepatobiliary Pancreat Sci. (2018) 25(1):41–54. 10.1002/jhbp.515 29032636

[34] Zhu QD Tao CL Zhou MT Yu ZP Shi HQ Zhang QY. Primary closure versus T-tube drainage after common bile duct exploration for choledocholithiasis. Langenbecks Arch Surg. (2011) 396(1):53–62. 10.1007/s00423-010-0660-z 20582601

[35] Zhang Z Ji H Chen G Hou Y. A comparative study of laparoscopic choledocholithotomy with primary suture and T-tube drainage. Medicine (Baltimore). (2024) 103(1):e36757. 10.1097/MD.0000000000036757 38181295 PMC10766256

[36] Hua J Lin S Qian D He Z Zhang T Song Z. Primary closure and rate of bile leak following laparoscopic common bile duct exploration via choledochotomy. Dig Surg. (2015) 32(1):1–8. 10.1159/000368326 25613528

[37] Ma X Cai S. The outcome and safety in laparoscopic common bile duct exploration with primary suture versus T-tube drainage: a meta-analysis. Appl Bionics Biomech. (2023) 2023:7300519. 10.1155/2023/7300519 36816756 PMC9929208

[38] Li L Zeng Z Li L Zhang J. Comparison of the therapeutic effects of three minimally invasive approaches for laparoscopic cholecystectomy combined with common bile duct exploration–a 5-year retrospective analysis. BMC Surg. (2024) 24(1):199. 10.1186/s12893-024-02490-4 38956622 PMC11218252

[39] Jiang Y Zhang J Li W Li L. Primary closure versus T-tube drainage after laparoscopic common bile duct exploration in patients with non-severe acute cholangitis. Updates Surg. (2022) 74(3):899–906. 10.1007/s13304-021-01214-0 34988916

[40] Zhuang L Li Y Zhang L Xu X Sun D Xi D A comparison of the therapeutic outcomes between primary duct closure and T-tube drainage after laparoscopic common bile duct exploration: a single-centre retrospective study. Wideochir Inne Tech Maloinwazyjne. (2023) 18(1):108–16. 10.5114/wiitm.2022.120672 37064551 PMC10091919

